# Genetic diversity and background pollen contamination in Norway spruce and Scots pine seed orchard crops

**DOI:** 10.48130/FR-2022-0008

**Published:** 2022-06-01

**Authors:** Alisa Heuchel, David Hall, Wei Zhao, Jie Gao, Ulfstand Wennström, Xiao-Ru Wang

**Affiliations:** 1 Department of Ecology and Environmental Science, Umeå Plant Science Center, Umeå University, Umeå SE-90187, Sweden; 2 CAS Key Laboratory of Tropical Forest Ecology, Xishuangbanna Tropical Botanical Garden, Chinese Academy of Sciences, Menglun 666303, Yunnan, China; 3 The Forestry Research Institute of Sweden (Skogforsk), Sävar SE-918 21, Sweden

**Keywords:** Genetic diversity, Mating structure, Parental contribution, Pollen contamination, Seed orchard, *Picea abies*, *Pinus sylvestris*.

## Abstract

Seed orchards are the key link between tree breeding and production forest for conifer trees. In Sweden, Scots pine and Norway spruce seed orchards currently supply ca. 85% of seedlings used in annual reforestation. The functionality of these seed orchards is thus crucial for supporting long-term production gain and sustainable diversity. We conducted a large-scale genetic investigation of pine and spruce orchards across Sweden using genotyping-by-sequencing. We genotyped 3,300 seedlings/trees from six orchards and 10 natural stands to gain an overview of mating structure and genetic diversity in orchard crops. We found clear differences in observed heterozygosity (*H*_O_) and background pollen contamination (BPC) rates between species, with pine orchard crops showing higher *H*_O_ and BPC than spruce. BPC in pine crops varied from 87% at young orchard age to 12% at mature age, wherease this rate ranged between 27%−4% in spruce crops. Substantial variance in parental contribution was observed in all orchards with 30%−50% parents contibuting to 80% of the progeny. Selfing was low (2%−6%) in all seed crops. Compared to natural stands, orchard crops had slightly lower *H*_O_ but no strong signal of inbreeding. Our results provide valuable references for orchard management.

## INTRODUCTION

Increasing demands on forest products drive industrial scale reforestation globally. In Sweden, more than 377 million seedlings of Scots pine (*Pinus sylvestris* L.) and Norway spruce (*Picea abies* (L.) Karst.) are planted every year, of which ca. 85% are supplied by seed orchards^[1]^. Seed orchards are artificial breeding populations containing elite or plus trees that have been selected from natural stands or testing trails on the basis of phenotypic desirability for producing genetically improved seeds for forest regeneration. In conifer tree improvement, seed orchards represent the key link between breeding programs and production forests by uniting elevated gain through artificial selection with high seed production from silviculture practice^[2]^. For conifer species that lack convenient clonal propagation, well-functioning seed orchards that incorporate both breeding gain and long-term diversity into their design are the most cost-effective method for sustainable forestry with enhanced gain^[3,4]^.

To produce seeds in a predictable manner while ensuring long-term forest harvesting capacity, seed orchards should meet certain requirements, e.g. balanced mating among parents, low levels of inbreeding, sufficient genetic diversity and minimum inflow of external pollen in seed crops^[2,5,6]^. Mating in seed orchards is expected to be random and each parent should contribute proportionally to the progeny to ensure a broad genetic base and the expected breeding gain. In reality, however, these expectations are rarely fully met. Due to a large variation in fecundity and flowering time among orchard parents, unbalanced contributions among parents to the progeny gene pool are commonly observed and can lead to increased relatedness and loss of genetic diversity in seed crops^[7−10]^. High genetic diversity in seed crops is important because it supports adaptation to changing climate and resilience of boreal forest ecosystems. Genetic diversity is a high priority in Swedish tree breeding programs^[11]^, and considerable effort is put into seed orchard design to optimize number of parents for maximum gain while maintaining high diversity^[6,12,13]^.

A substantial proportion of breeding gain can, however, be lost as a result of background pollen contamination (BPC) from adjacent unimproved stands. Pine and spruce seed orchards in Sweden are open-pollinated and complete isolation from natural conspecific stands where Scots pine and Norway spruce forests make up more than 80% of the land cover is impossible. Background pollen contamination of seed orchards reduces the genetic gain of the crop by a factor proportional to the BPC rate^[14]^, and thus represents a major concern for orchard management in Nordic countries. Examination of BPC rates has been a focus of most seed orchard investigations, and large variations in BPC are found in Scots pine orchards, ranging from 8% to 59%^[15,16]^. While BPC in Scots pine orchards is more intensively investigated, e.g. Torimaru et al.^[8]^ and Funda et al^[17]^, less is known about BPC and its dynamics among Norway spruce orchards due to infrequent flowering in this species. The few available studies on spruce orchards reported BPC rates ranging from 10% to 70%^[18−20]^. This large variation can be due to many factors, e.g. orchard age, location, internal pollen production, plantation density and orchard management practices, but also marker resolution. Despite decades of research, comparative evaluation of the differences in mating system between pine and spruce orchards is still lacking. Investigations of BPC rates and genetic compositions of orchard crops can guide management actions to reach the expected gain and diversity and further assist dynamic deployment of seedlots to suitable climate zones to optimize forest production and economy^[21]^. This is particularly relevant for the advanced Swedish seed orchards where the expected breeding gain is as high as 25%^[4]^.

Here we present a large-scale genetic study of both Scots pine and Norway spruce seed orchards and natural stands. We genotyped 3,300 seedlings and parent trees from six orchards and 10 natural stands across Sweden using genotyping-by-sequencing (GBS). Our objectives were: 1) to characterize mating structure, diversity and BPC in both pine and spruce orchards; 2) to assess whether and how BPC varies between species and orchards of different ages and parental compositions; 3) to compare genetic diversity between orchard crops and reference natural populations to understand whether there is a reduction in diversity. The findings from this study provide new insights into the mating systems of the two species and serve as a useful reference for seed orchard management.

## MATERIALS AND METHODS

### Study system

**Figure 1:**
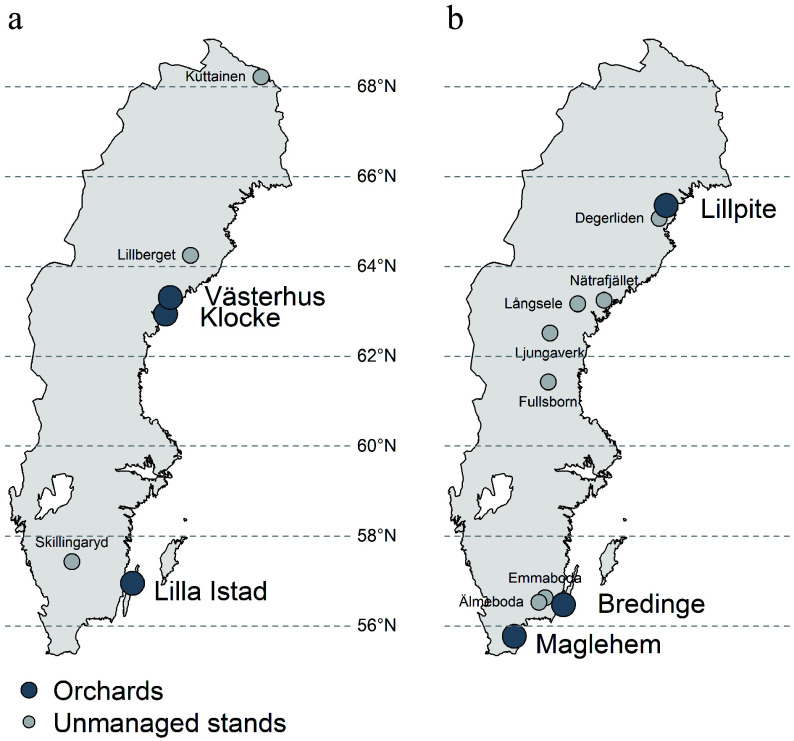
Map of seed orchards and natural stand locations sampled for this study. (a) Scots pine, (b) Norway spruce.

We selected three pine and three spruce seed orchards from the north and south of Sweden and three pine and seven spruce natural stands as references for this study (Fig. 1, Table 1). These orchards are established at different times (1957−1991) and differ in size and parental composition (Table 1). Seed orchard Västerhus is designed following a linear deployment strategy in that clones with higher breeding values are proportionally more represented. Other orchards follow a partial randomization setup. All orchard parents are plus-trees representing different cycles of selection, e.g. parents in Klocke, Lillpite, Maglehem are the 1^st^ round of selection from natural stands, while Lilla Istad, Västerhus, Bredinge are the 2^nd^ round selection based on progeny test results. In this regard, all orchards are first generation orchards with phenotypically selected plus trees from natural stands or backward selected plus trees after progeny testing. The Klocke orchard contains the most northern selections with the origins ranging from 66.4° to 68.5° N, Västerhus 61.9°−65.1° N, and Lilla Istad 56.8°−61.3° N. The spruce orchards are more mixed; Lillpite has parents originating from 63.6° to 67.6° N, Bredinge has parents from Romania, Slovakia, Poland, Belarus, Latvia and southern Sweden, and Maglehem are based on phenotypically selected trees in southern Sweden that were planted in the beginning of the 20^th^ century from German/continental Europe seed sources. The deployment areas of each orchard are shown in Supplemental Fig. S1.

**Table 1:** Summary of samples included in this study.

Orchard & stand name	Type of material	Latitude	Longitude	Year orchard established	Orchard size (ha)	Orchard age of the crop	Orchard crop size (kg/ha)	No. parents in orchard	No. parents genotyped	No. seedlings genotyped
**Scots pine**										
Lilla Istad orchard	Crop 2007	56°57' N	16°48' E	1982	21	25	4.4	40	39	314
Klocke orchard	Crop 1985	62°56' N	18°21' E	1970	16	15	0.8	66^1^	63	215
Klocke orchard	Crop 1996					26	2.8			293
Klocke orchard	Crop 2008					38	1.1			301
Västerhus orchard	Crop 2014	63°18′ N	18°33′ E	1991	13.7	23	5.4	28	28	299
Västerhus orchard	Rootstock								67	
Skillingaryd	Natural stand	57°43' N	14°02' E							50
Kuttainen	Natural stand	68°22' N	22°48' E							50
Lillberget	Natural stand	64°17' N	19°33' E							50
Total									197	1,572
**Norway spruce**										
Lillpite orchard	Crop 1983	65°21' N	21°10' E	1963	10	20	38.0	36	35	231
Bredinge orchard	Crop 2000	56°28' N	16°25' E	1983	12	17	4.4	204^2^	146	431
Maglehem orchard	Crop 1993	55°46' N	14°10' E	1957	5	36	21.0	36	32	137
Maglehem orchard	Crop 2000					43	44.0	32^3^	30	304
Älmeboda	Natural stand	56°38' N	15°15' E							27
Emmaboda	Natural stand	56°37' N	15°31' E							39
Fullsborn	Natural stand	61°25' N	15°42' E							17
Ljungaverk	Natural stand	62°31' N	15°48' E							26
Långsele	Natural stand	63°10' N	17°4' E							36
Nätrafjället	Natural stand	63°10' N	18°14' E							11
Degerliden	Natural stand	65°04' N	20°51' E							29
Total									243	1,288
^1^ Six genotypes were planted with 1 or 2 ramets at establishment, some of them were lost at later age. ^2^ Genetic thinning in Bredinge during 2012−2013 removed a section containing 58 parents. ^3^ Genetic thinning in Maglehem in 1994 reduced the number of genotypes and changed ramet frequency among remaining genotypes.										

Cones were harvested in bulk from orchards and natural stands and seeds were randomly sampled from each seedlot. Commercial forest companies were tasked to harvest cones in natural stands with the requirement that cones from a minimum of 100 trees were collected. These natural stands are important references and need to be representative of locally adapted material for Skogforsk's yearly hardiness evaluation of commercial crops and references for comparison to improved material.

A collection of seeds harvest in a particular orchard in a particular year is called a crop (Table 1). From two orchards, Klocke and Maglehem, we sampled multiple crops at different orchard ages to understand the genetic changes in seed crops along orchard age progression (Table 1). Seeds were germinated in a greenhouse and needles were collected from the seedlings for genotyping. Needles were also sampled from all parents in each orchard to establish their genotypes to facilitate parentage assignment of seedlings. In one orchard, Västerhus, we identified 67 rootstock which had grown into mature trees after the graft died out, thus becoming an unwanted potential pollen source within the orchard. We included rootstock trees in the genotyping to obtain a more accurate estimate of external pollination rate in this orchard. We analyzed five Scots pine and four Norway spruce crops with a sample size of 137−431 seedlings per crop (Table 1). For each natural stand, 11−50 seedlings were sampled. In total, 1,769 pine and 1,531 spruce samples (including orchard parents) were genotyped (Table 1).

### Genotyping-by-sequencing (GBS)

Genomic DNA was extracted from needles using the E-Z 96 Plant DNA Kit (OMEGA, USA) and quantified using Qubit® DNA HS (High Sensitivity) Assay Kits (Invitrogen, Thermo Fisher Scientific™, USA) on a Synergy HTX multi-mode reader (BioTek, USA). GBS libraries were prepared following the procedure of Pan et al.^[22]^. Briefly, 200 ng DNA from each individual were digested with *Pst*I restriction enzyme (New England BioLabs, UK) and ligated to an individual barcode. Equal amounts of digested DNA from 300 individuals, each with unique barcodes, were pooled and purified with QIAquick PCR Purification kit (QIAGEN, Germany). The resulting pool of DNA fragments were PCR amplified with initial denaturation at 98 °C for 30 s, followed by 15 cycles of denaturation at 98 °C for 10 s, annealing at 65 °C for 30 s, elongation at 72 °C for 20 s, and final elongation at 72 °C for 2 min. The PCR products were purified and separated on 2% pre-cast agarose gel (E-Gel, Invitrogen, Thermo Fisher Scientific, Israel). Fragments in the size range of 350–450 bp were excised from the gel and extracted with QIAquick Gel Extraction kit (QIAGEN, Germany) and quantified with PicoGreen dsDNA kit (Invitrogen, Thermo Fisher Scientific, USA). Paired-end sequencing was performed on Illumina HiSeq 2500 or on Illumina HiSeqX Ten by NovoGene (Hong Kong, China).

### Processing sequence reads

Adapter sequences and low‐quality bases (Phred quality < 20) were removed from the tail of each read using Trimmomatic v0.36^[23]^. The clean reads were demultiplexed using the process_radtags module of Stacks v1.46 with disable_rad_check parameter^[24]^. Reads shorter than 41 bases were discarded. The first five bases of each resulting read, which is the *Pst*I recognition site, were removed with the FASTX-Toolkit (http://hannonlab.cshl.edu/fastx_toolkit/indexhtml). Paired reads were mapped against the reference genomes of *Pinus taeda* v1.01^[25]^ and *Picea abies*^[26]^ for Scots pine and Norway spruce, respectively, using the BWA-MEM algorithm^[27]^ with default parameters. Variants were called with SAMtools and BCFtools pipeline^[28]^. We removed SNPs with mapping quality of less than 30 and SNPs within 5 bp around indels and in repetitive regions. Individuals with more than 40% missing data were removed. Genotypes with sequencing depth < 5 or genotype quality < 20 were masked as missing. SNPs with a minor allele frequency (MAF) less than 0.05, missing rate > 40%, heterozygosity > 70% or allele number > 2 were also removed.

### Genetic diversity

We estimated observed (*H*_O_) and expected heterozygosity (*H*_E_) and inbreeding coefficients (*F*_IS_) for each seed crop and natural stand with the R package 'hierfstat'^[29]^. During the analyses, we observed a reduction in heterozygosity with decreasing breadth of coverage and thus used only those samples with a high breadth of coverage (≥ 80% of total SNP number) for heterozygosity and *F*_IS_ calculations. The 95% confidence interval for *F*_IS_ was estimated by jackknifing a sample size of (total SNP number)/60 (which corresponds to approximately 100 per resampling) for 100,000 resamples in the function 1 – *H*_O_/*H*_E_. Differences in diversity measurements between species and seed sources (i.e. orchard crop or natural stand) were examined by fitting a linear model with generalized least squares implemented in the *gls* function of the 'nlme' package in R^[30]^, which allows for heteroscadacity among categories.

To evaluate whether there are allelic shifts between seed orchard crops and orchard parents and natural stands, we performed principle component analysis (PCA) on all the genotyped trees and seedlings of each species. Because different populations and crops were sequenced in different libraries, to diminish batch effect in the combined sample set, we imposed more stringent SNP filtering allowing missing rate of only 10% for this analysis. We ran PCA using the R-package 'pcadapt'^[31]^ for each orchard including natural stands as reference. Because the sample differences of each subset and the pcadapt's default MAF cut-off 0.05, the number of SNPs used in PCA for each subset was slightly different (Lilla Istad 2,038, Västerhus 2,270, Klocke 1,741, Maglehem 1,175, Bredinge 1,260, and Lillpite 1,154).

### Parentage assignment

To assess the effective number of parents (*N*_ep_) and subsequently calculate the rate of BPC in orchard crops, we performed parentage reconstructions for orchard progenies given the parental genotypes in each orchard and the allele frequencies of conspecific natural reference stands. Parentage reconstructions were executed using Ritland's estimator^[32]^ in the 'related' R package^[33]^, which is based on a methods of moments estimator applied to a bi-allelic genotype score matrix^[32]^. Theoretically, the relatedness estimate for parent-offspring should be exactly 0.5, however, due to sequencing errors and false heterozygote and homozygote calls at reduced coverage, the estimate varies and is often reduced in GBS data^[34,35]^. We also applied pairwise genotype score correlation among samples (genotype score 0, 1, or 2 for each locus, where 0 is homozygote for the reference allele, 2 homozygote for the alternate allele and 1 is the heterozygote genotype) as a neutral control of similarities. The thresholds for Ritland's estimator and genotype score correlation threshold for assignment as parent-offspring pairs were adjusted for each genotype pair relative to all other sample comparisons according to Hall et al.^[34]^. Based on pairwise relatedness estimates between seedlings, the assigned parent-offspring pairs were then used to determine whether external pollen donors were unique.

Effective number of parents, which in this setting represents the effective population size of a crop, was estimated under two different conditions: first, based on the orchard parents only and their proportional contribution to the crop (*N*_ep_); second, all unique parents that contributed to the orchard crop including unknown pollen donors (*N*_ep2_). *N*_ep2_ estimates the actual effective number of unique genotypes that contributed to the crop. Effective number of parents was estimated based on the function of the effective number of types^[36]^ using sampling-bias corrected estimate^[37]^:

$  {N}_{{e}_{p}}=\frac{{\left(n-1\right)}^{2}}{\sum _{i=1}^{{N}_{p}}{P}_{{suc}_{i}}^{2}\left(n+1\right)\left(n-2\right)+3-n} $

where *n* is the number of seedlings sampled, *N*_*p*_ is the number of parents, and $P_{suc_i} $ is the relative reproductive success of the *i*-th parent. Calculations were performed both with and without external pollen resources. For each external pollination event, the relationship of the seedling was compared to other seedlings to define it as unique or not.

Unique pollen donors of a progeny not matching any genotyped orchard parents were regarded as external pollination events. However, if multiple pollination events from an unknown pollen donor occurred in a group of closely related seedlings, we assumed that this pollen donor was more likely to be a un-genotyped orchard parent. This kind of event could occur in orchards that had contained unique parental genotypes at the time of seed crop pollination (see crop date, Table 1) but that had been lost as a result of thinning or die-off prior to the genotype sampling of the parents in this study. For example, an entire section of the Bredinge orchard that had harbored unique genotypes was removed in 2011−2012 (parents not genotyped then), although these parents could have contributed to the Bredinge crop 2000 studied here.

Finally, we examined the Spearman correlations *ρ* between diversity parameters (*H*_O,_ *H*_E_ and *F*_IS_) and mating system parameters (BPC, number of parents in orchard *N*_ep_, and the variation in logit transformed, to control for heteroscedasticity, of relative reproductive success_*,*_ Var(ln(*P*_*suc*_/(1 – *P*_*suc*_))) and orchard age. We further examined the effect size of the mating system parameters on diversity using the *gls* model to understand the impact of each factor on seed crop quality. Although putative relationships between parameters are expected, the ability to predict crop diversity based on the mating parameters from models would be of interest.

## RESULTS

### Single nucleotide polymorphisms (SNPs)

Sequence reads were first grouped into two datasets by species and filtered separately. Before SNP filtering, we obtained 23 million sites for pine and 44 million sites for spruce. Bases with a coverage of five reads or more had a mean depth of 48 and 42, and median depth of 8 and 10 for Scots pine and Norway spruce, respectively. After the removal of indels, sites with low sequencing depth, and genotypes with high rates of missing data and multi-allelic sites, 21,626 and 15,581 sites were kept for pine and spruce, respectively. The removal of low frequency alleles (MAF < 0.05) reduced the number of sites further to 6,736 SNP in pine and 5,622 SNP in spruce. A total of 3,300 samples were successfully genotyped of which 1,769 represent pine and 1,531 represent spruce (Table 1).

### Genetic diversity

**Figure 2:**
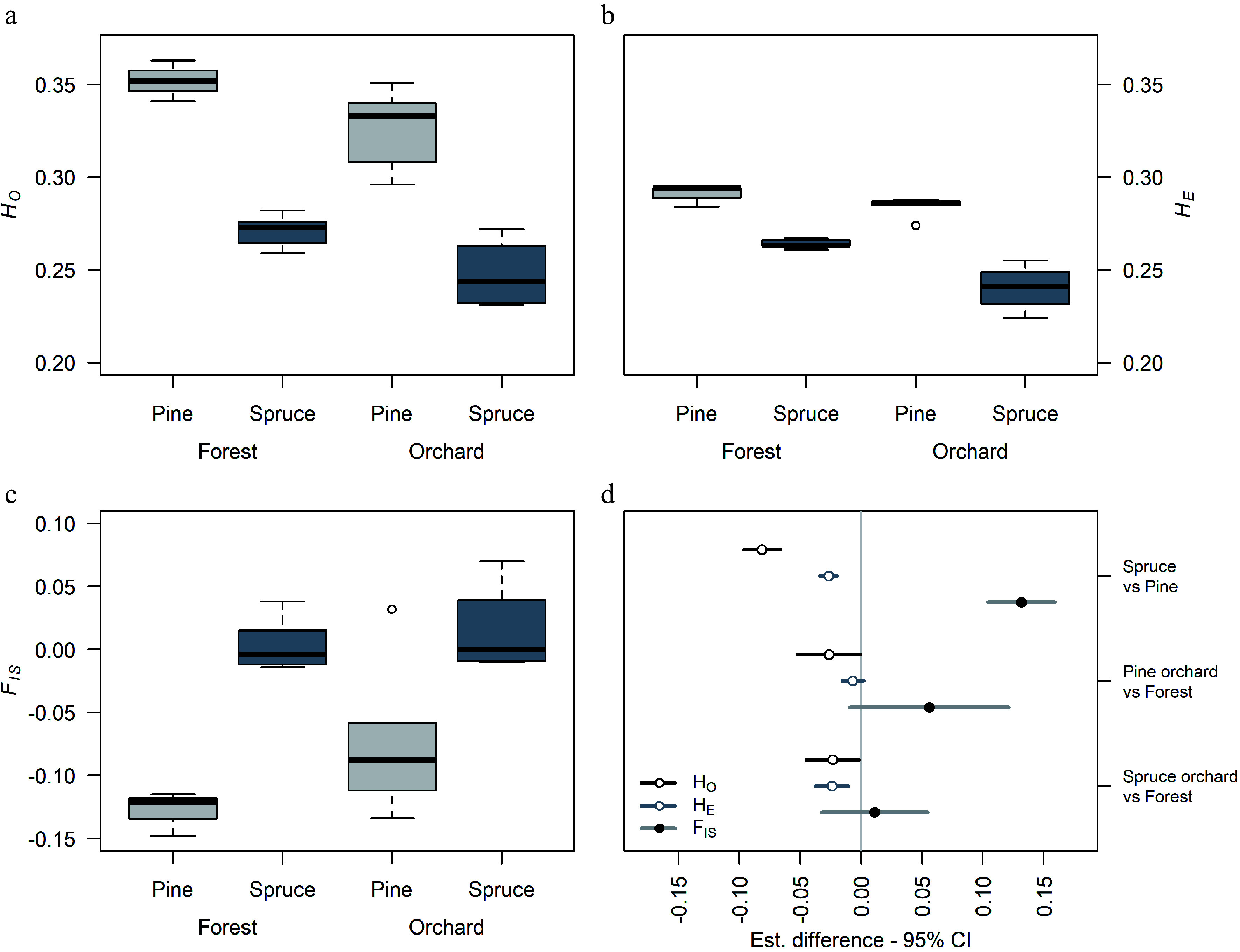
Comparison of genetic diversity in Norway spruce and Scots pine seed orchard crops and natural stands. (a) Variation in observed heterozygosity *H*_O_, (b) expected heterozygosity *H*_E_, (c) inbreeding coefficient *F*_IS_ in orchard crops and natural stands, and (d) the general least squares (*gls*) comparison between species and seed source within species in diversity estimates. Bar length indicates 95% confidence interval (CI).

High genetic diversities were found in Scots pine orchard crops and natural stands, with observed heterozygosities *H*_O_ ranging from 0.296 to 0.363 (Table 2). On average, natural stands of Scots pine had slightly higher values of *H*_O_ than orchard crops, and the lowest value was found in the 2008 crop from Klocke orchard. Norway spruce had lower *H*_O_ than Scots pine, with values that ranged between 0.231 and 0.282. Similar to the results in Scots pine, natural stands of Norway spruce had higher *H*_O_ than orchard crops, with the lowest *H*_O_ found in the Maglehem 2000 crop and Lillpite 1983 crop (Table 2). Differences in *H*_O_ between species was on average 0.081 lower than pine (*p* < 0.0001) (Fig. 2a & d) and that *H*_O_ in orchard crops was significantly lower (0.026 and 0.023 for pine and spruce, respectively) than *H*_O_ in natural stands (Fig. 2d).

**Table 2:** Results of genetic diversity and parentage analyses. Genetic diversity in orchard crops and natural stands were measured by observed *H*_O_ and expected *H*_E_ heterozygosity and inbreeding coefficient *F*_IS_.

Orchard crop, natural stand	Sample size*	*H*_O_ (± SE)	*H*_E_ (± SE)	*F*_IS_ (95% CI)	Both parents assigned	BPC	Selfing	Not assigned	*N*_ep_	*N*_ep2_
Scots pine										
Lilla Istad 2007	314 (261)	0.351 ± 0.001	0.288 ± 0.0006	−0.134 (−0.189−0.083)	169 (53.9%)	136 (43.3%)	5 (1.6%)	9	26.6	46.1
Klocke 1985	215 (199)	0.333 ± 0.0012	0.287 ± 0.0008	−0.088 (−0.145−0.034)	24 (11.2%)	186 (86.5%)	6 (2.8%)	5	29.8	122.0
Klocke 1996	293 (212)	0.308 ± 0.0011	0.274 ± 0.0007	−0.058 (−0.113−0.007)	251 (85.7%)	35 (11.9%)	17 (5.8%)	7	51.5	59.5
Klocke 2008	301 (276)	0.296 ± 0.0009	0.285 ± 0.0005	0.032 (−0.031−0.092)	152 (50.5%)	145 (48.2%)	16 (5.3%)	4	36.7	68.7
Västerhus 2014	299 (277)	0.340 ± 0.0009	0.286 ± 0.0005	−0.112 (−0.166−0.061)	202 (67.6%)	93 (31.1%)	9 (3.0%)	4	16.8	24.0
Skillingaryd	50 (45)	0.341 ± 0.0057	0.284 ± 0.0035	−0.121 (−0.181−0.064)						
Kuttainen	50 (47)	0.352 ± 0.0057	0.294 ± 0.0033	−0.115 (−0.181−0.051)						
Lillberget	50 (49)	0.363 ± 0.0055	0.294 ± 0.0031	−0.148 (−0.210−0.088)						
Sum	1,572 (1,366)				798	595	53	29		
Norway spruce										
Lillpite1983	231 (160)	0.233 ± 0.0012	0.243 ± 0.001	0.070 (0.018−0.121)	209 (90.5%)	20 (8.7%)	7 (3.0%)	2	23.6	26.0
Bredinge 2000	431 (347)	0.272 ± 0.0006	0.255 ± 0.0004	−0.010 (−0.060−0.036)	313 (72.6%)	116 (26.9%)	11 (2.6%)	2	65.0	89.7
Maglehem1993	137 (127)	0.254 ± 0.0017	0.239 ± 0.0013	−0.008 (−0.066−0.048)	130 (94.9%)	5 (3.6%)	5 (3.6%)	2	25.1	26.2
Maglehem 2000	304 (259)	0.231 ± 0.0008	0.224 ± 0.0006	0.008 (−0.041−0.055)	277 (91.1%)	20 (6.6%)	7 (2.3%)	7	20.9	22.4
Älmeboda	27 (22)	0.273 ± 0.0098	0.263 ± 0.0072	−0.004 (−0.069−0.059)						
Emmaboda	39 (36)	0.282 ± 0.0058	0.267 ± 0.0042	−0.014 (−0.073−0.043)						
Fullsborn	17 (17)	0.260 ± 0.0127	0.263 ± 0.0099	0.029 (−0.042−0.102)						
Ljungaverk	26 (26)	0.259 ± 0.008	0.263 ± 0.0063	0.038 (−0.027−0.103)						
Långsele	36 (34)	0.277 ± 0.0063	0.265 ± 0.0047	−0.010 (−0.072−0.052)						
Nätrafjället	11 (10)	0.269 ± 0.024	0.261 ± 0.0183	−0.014 (−0.095−0.07)						
Degerliden	29 (24)	0.275 ± 0.0092	0.267 ± 0.0069	0.001 (−0.067−0.069)						
Sum	1,288 (1,062)				929	161	30	13		
BPC – Background pollen contamination. *N*_ep_ – effective no. of parents from orchard. *N*_ep2_ – effective no. of parents including external pollen donors. Selfing belongs to the category 'Both parents assigned' * Sample size within parenthesis are the number of samples with high breadth of coverage (≥ 80% of the total number of SNPs) used for heterozygosity and *F*_IS_ estimates										

Expected heterozygosity *H*_E_ was less variable between seed sources (i.e. orchard crop or natural stand) within each species, but the difference between species was significant (*p* < 0.0001, Fig. 2b). Spruce had lower *H*_E_ on average than pine, and spruce crops had lower *H*_E_ than natural stands. However, no such significant reduction was observed in pine orchard crops compared to natural stands (Fig. 2b, d). Because, spruce had significantly lower diversity estimates than pine, we observed significantly greater (0.132) *F*_IS_-values overall in spruce than pine on average (*p* < 0.0001). On average, however, we do not see an elevation of inbreeding coefficients in crops of either species compared to their respective natural stands (Fig. 2d). In general, most sample groups in both species had *F*_IS_ values close to zero, particularly in spruce (Table 2, Fig. 2c), suggesting panmixia.

**Figure 3:**
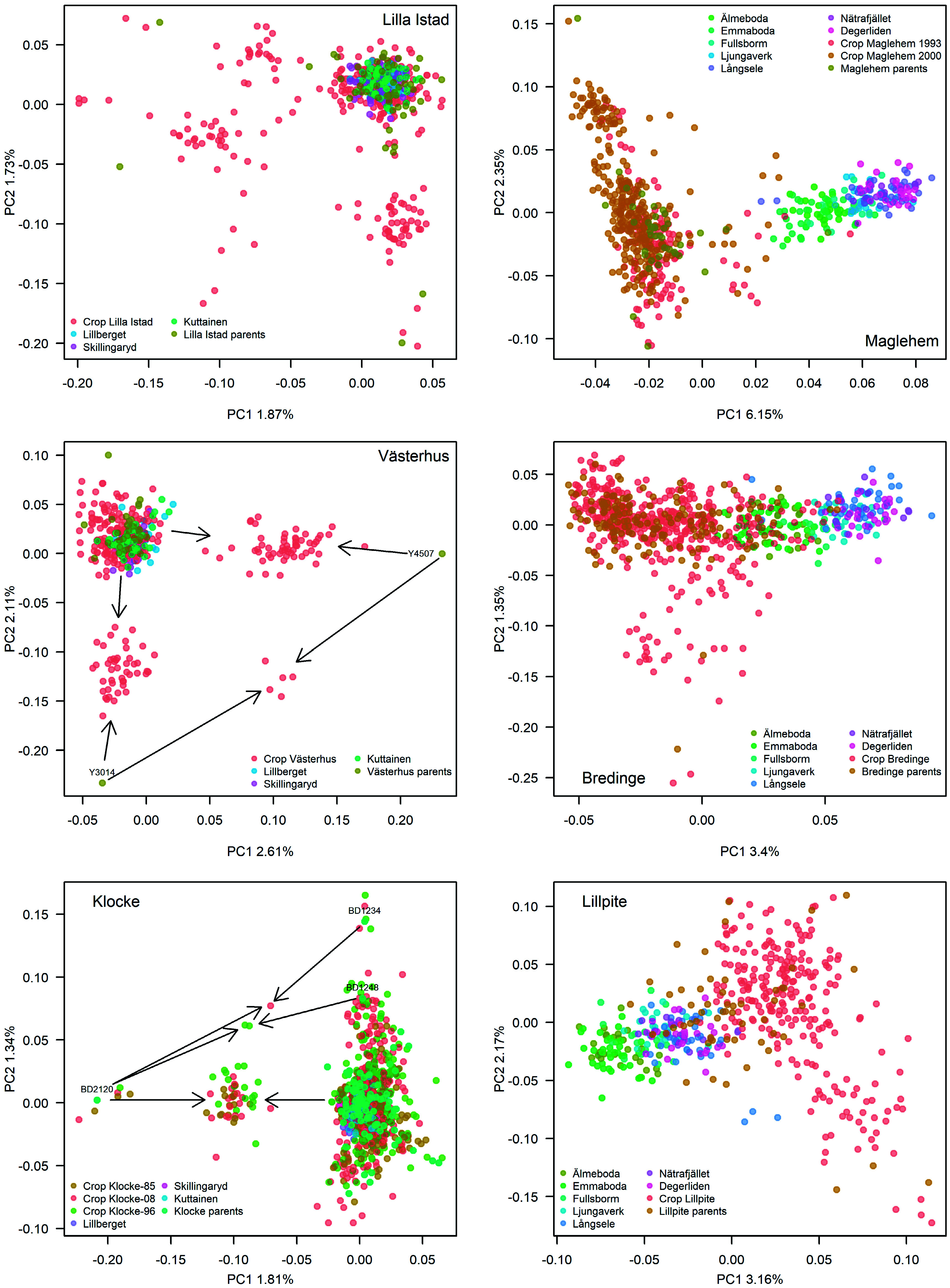
PCA plots of genetic variation of each orchard crop(s) and parents in comparison to natural stands. Left panels depict the pine orchards while the right panels depict the spruce orchards. Where arrows are depicted, they point to a group of offspring, which the particular parent has contributed to. In Klocke, parent BD2120 and BD1234 each has six and five offspring from self-pollination, shown as dots around each parent.

**Figure 4:**
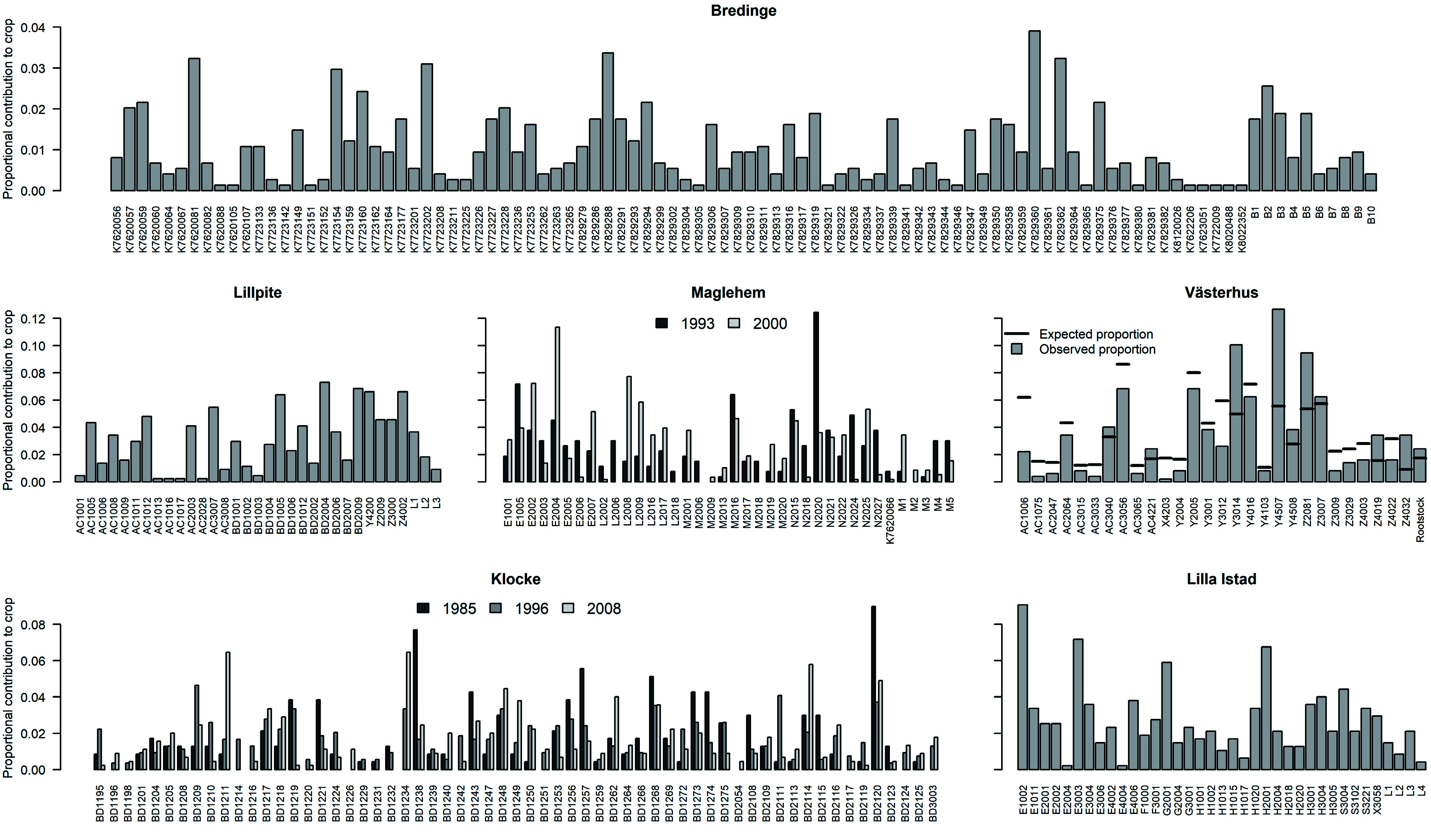
Parental contribution to each orchard crop. Parent IDs are on the x-axis.

The PCA on genetic diversity in orchard crops, parents and natural stands revealed a distinct difference between spruce and pine, where spruce displayed a population structure effect along the first PC axis but pine did not (Fig. 3). Pine on the other hand displayed relatedness on both PC-axis 1 and 2, while spruce showed relatedness on PC2 only. The pine orchard crops in general overlapped completely with the natural stands, and little genetic differentiation was detected between crops, parents and reference stands. A few clusters on the Västerhus and Klocke PCA plots were driven by parents dominating the crop of each orchard. In Klocke, the most successful parent BD2120 (Fig. 4) drove PC1 and the second most successful parent BD1234 PC2; these two parents also produced six and five offspring from self-pollination respectively, creating a small cluster around each (Fig. 3). In Västerhus, parents Y4507 and Y3014 drove PC1 and 2, respectively. The PCA pattern in spruce orchard crops reflect their parents' origins and thus a slight difference in allele frequencies relative to the reference natural stands. This is particularly visible in Bredinge and Maglehem, in which parent populations shifted from natural stands in accordance with their central Europe origins, and the slight differentiation among the reference stands reflect their positions on a south to north gradient.

### Mating structure and BPC in seed orchards

We found no relatedness among orchard parents, and performed parentage reconstruction on 2525 seedlings from nine crops and six orchards. A single parent was identified for 2,483 seedlings (1,393 pine and 1,090 spruce), and both parents were identified for 1,727 seedlings (798 pine and 929 spruce, Table 2). However, 2−9 seedlings in each seed crop (i.e. 0.5%−2.9%) could not be assigned to any putative parents in their respective orchard (Table 2). These likely represent seeds from non-orchard sources (i.e. contamination), or are individuals with sequence coverage too low to be reliably assigned. Self-fertilization rate in each crop was low and ranged from 1.6%−5.8% in pine and 2.3%−3.6% in spruce. Spruce crops were less variable in selfing rate than pine crops.

Seeds with only one matching parent in an orchard were regarded as being sired by external background pollen if this source was unique. The range of BPC estimates was large in pine orchard crops where both extremes occurred among Klocke crops, from 86.5% at orchard age 15 (crop 1985) to 11.9% at age 26 (crop 1996). The third Klocke crop (2008) at age 38 had a BPC rate of 48.2% (Table 2). Klocke orchard was thinned in 2004 at age 34, which likely reduced the internal pollen production and opened up corridors for inflow of background pollen. The crop from Lilla Istad (age 25) had 43.3% while the Västerhus crop (age 23) had 31.1% BPC.

Spruce orchard crops, on the other hand, generally had much lower BPC. The lowest estimate was 3.6% in Maglehem 1993 crop (age 36) and the highest in the Bredinge 2000 crop with 26.9% (age 17). However, 58 parental genotypes (28%) from the Bredinge orchard have not been genotyped because they were removed by thinning in 2011–2012, before the collection of parents for this study was made. These parents very likely contributed to crops before 2011, but would be identified as BPC. Thus the BPC rate in Bredinge 2000 crop is inflated and we expect the true BPC of this crop to be much lower. The remaining two crops, Maglehem 2000 and Lillpite 1983, had 6.6% and 8.7% BPC, respectively (Table 2).

The effective numbers of parents in each crop were estimated without (*N*_ep_) and with external pollination (*N*_ep2_). *N*_ep_ reflects mating among orchard parents. Unbalanced gametic contribution to the seed crop makes *N*_ep_ lower than census number of parents, which was observed in all orchard crops. The most marked difference occurred in the Bredinge 2000 crop (Table 2), which again could result from the large proportion of ungenotyped orchard parents. The number of effective parents *N*_ep2_ includes the external paternal contribution and is thus larger than *N*_ep_. While all pine crops showed a substantially elevated *N*_ep2_ estimate, only the Bredinge crop had a noticeably elevated *N*_ep2_ among the spruce crops due to its higher BPC estimate.

**Figure 5:**
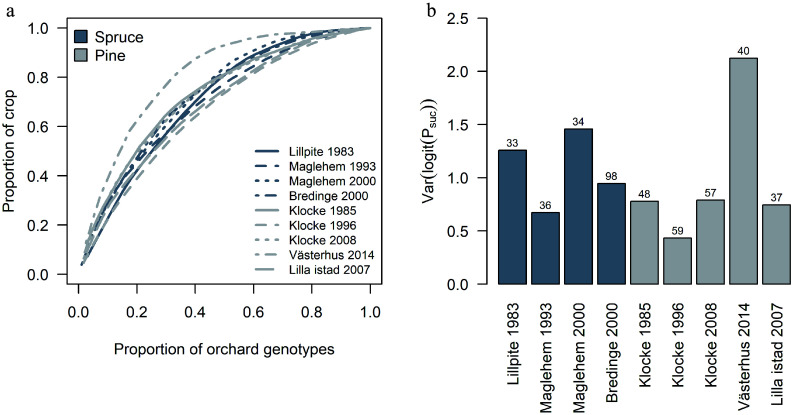
Variation in parental contribution among orchard crops. (a) The cumulative sums of orchard parents' contribution to each crop. (b) The variation in parents' contribution among orchards crops. Numbers above bars indicate the number of identified parents (and rootstock for Västerhus) within the orchard that contributed to the crop.

Reproductive success among parents within each orchard varied greatly in each seed crop (Fig. 4). In Västerhus 2014 crop, 32% of the parents contributed to 80% of progeny (Fig. 5a), reflecting a large variance in reproductive success among parents (Fig. 5b). Västerhus is designed as a linear deployment orchard, with intentional unequal representation of parents. We compared the observed gametic contribution to the expected and found that the two values rarely matched (Fig. 4). Spruce orchards showed similar levels of variance in parental contributions with Bredinge 2000 having the lowest variance. With the exception of Västerhus, around 50% of the parents contributed to 80% of the progeny in all orchards (Fig. 5a). In the Västerhus orchard crop we found contributions from the overgrown rootstock, with 12 rootstock genotypes detected as parent for one seedling each (Fig. 4). We also detected extra parents in the seed crops from four other orchards (Bredinge, Lillpite, Maglehem, and Lilla Istad), which likely represent ungenotyped orchard parents that could have come from rootstock and/or supplemental planting of additional genotypes.

We expected some statistical associations between mating system parameters and genetic diversity estimates (Table 3). We found statistically significant Spearman's ρ between *H*_O_ and BPC (*ρ* = 0.68, *p* = 0.042), BPC and total effective number of parents *N*_pe2_ (*ρ* = 0.78, *p* = 0.013). Although we detected correlations between these parameters, none of the mating system parameters used in the *gls* modeling were able to predict the *H*_O_ of the orchard crops significantly well. Even though we analyzed an unprecedented number of crops and orchards in this study, there is still a need to increase sample sizes to infer how orchard design and mating parameters affect the genetic diversity of crops in each species.

**Table 3:** Spearman´s rank correlation between diversity and mating system parameters across orchard crops for both species. Significant ρ are in bold.

	*H*_O_	*H*_E_	*F*_IS_	BPC	Selfing	*N*_ep_	*N*_ep2_	Age	Census no. parents
*H*_E_	0.94***								
*F*_IS_	−0.88**	−0.66							
BPC	0.68*	0.74*	−0.47						
Selfing	−0.01	0.19	0.27	−0.13					
*N*_ep_	0	0.09	0.11	0.01	0.34				
*N*_ep2_	0.39	0.48	− 0.2	0.78*	0.09	0.57			
Age	−0.41	−0.42	0.34	−0.44	0.25	−0.31	−0.53		
Census no. parents	0.05	0.14	0.07	0.13	0.18	0.93***	0.61	−0.36	
*Var*(logit(*P*_*suc*_))	−0.05	−0.12	−0.05	−0.14	−0.40	−0.53	−0.47	0.00	−0.28
Significance level * *p *< 0.05, ** *p *< 0.01, *** *p *< 0.0001 *H*_O_ – observed heterozygosity; *H*_E_ – expected heterozygosity; *F*_IS_ – inbreeding coefficient; BPC – background pollen contamination; *N*_ep_ – effective no. of parents from orchard; *N*_ep2_ – effective no. of parents including external pollen donors; *P*_*suc*_ – relative reproductive success									

## DISCUSSION

### Genetic diversity in orchard crops

We detected significant differences in observed heterozygosity (*H*_O_) between the two study species. Both orchard crops and natural stands of Scots pine exhibited higher levels of *H*_O_ than those of Norway spruce (Fig. 2). Such distinct differences in diversity between the two species imply different demographic histories and mating system dynamics. Early studies using nuclear SSR markers to compare diversity in these two species were inconclusive^[38,39]^. In this study, > 5,000 loci were sampled in each species. Although 5,000 loci still represent only a small proportion of the mega-genomes of both species, this larger genomic sampling likely captured a more reliable estimate of the genetic diversity for comparing the two species.

We detected a small but significant decrease of *H*_O_ in orchard crops compared to natural stands. This was expected because orchards' parents only provide a limited gene pool. Although significant, the impact should be rather limited over time in orchard crops. In pine, varying genetic contribution of background pollen and strong purifying selection^[40]^ should facilitate assisted migration and faster adaptation. Spruce on the other hand is relatively stable in BPC-rates that result in predictable diversity estimates that can be accounted for with status number estimates during the orchard design^[41]^. We did not, however, detect significant inbreeding in orchard crops; all crops had *F*_IS_ values that were either negative or close to zero in both pine and spruce. Self-fertilization was low (2%−6%) in all crops in accordance with earlier estimates of selfing rates in orchards of these species^[8,17,19]^. We found slightly elevated selfing in the Klocke 1998 and 2000 crops (5%−6%). The parents in this orchard originated from the northern limit of the Scots pine distribution range, and are expected to have an increased proportion of viable self-pollinated seeds^[42,43]^. Although speculation, it is possible that the elevated selfing rate in the Klocke orchard crops is the result of increased selfing tolerance, resulting in larger proportions of viable seeds after self-fertilization^[44,45]^.

We found an expected correlation of *H*_O_ with BPC and *N*_ep2_, but no significant correlation between *H*_O_ or BPC and census number of parents in each orchard. In pine orchard crops, we saw a 16%−300% increase in effective number of parents (*N*_ep_ vs. *N*_ep2_) due to external pollination, with the Klocke 1985 crop having the highest values. The corresponding increases in spruce crops were 4%−38%, illustrating a differential impact of BPC on the diversity of orchard crops in the two species, and likely due to the more even BPC rates among spruce crops.

To gain a better view on whether there is allelic shift or genetic differentiation between orchard crops and parents and natural stands, we performed PCA on each species including all these categories of samples (Fig. 3). We see a distinct south-north gradient in allele frequencies in spruce in agreement with earlier findings on two genetic clusters in Norway spruce in Sweden^[46,47]^. We captured this structure in the Maglehem and Bredinge parents with origins from central and eastern Europe. With the low rates of BPC observed, spruce orchard crops reflect the orchard parents' allele frequencies rather than those surrounding the orchard. Scots pine on the other hand show no such patterns. Higher BPC rates in pine orchards and non-detectable population structure imply much greater gene-flow in Scots pine than Norway spruce, which resulted in no visible allele frequency shift between orchard progeny and natural stands in pine.

Reduction of diversity in orchard crops has been a concern of tree breeders, and various strategies have been implemented for orchard design to ensure both gain and diversity^[41,48,49]^. Our results show that in the 1^st^ generation seed orchards which are established with unrelated parents, diversity loss is not a major issue especially with the contribution of BPC, and that the orchards function reasonably well with respect to diversity and inbreeding. For outcrossing conifers like Scots pine and Norway spruce, first generation seed orchards with moderate number of parents are able to sustain genetic diversity. The performance of seed crops with varying degrees of BPC requires further evaluation to assess the realized breeding gain and adaptation to climate.

### Mating structure and BPC in pine and spruce orchards

The numbers of SNPs recovered in this study were well above the number needed to carry out precise parentage reconstruction^[34]^, which aided estimations of BPC, selfing and effective number of parents. Because no elevated relatedness was observed among orchard parents, the separation of the parent-offspring from unrelated relationships was straightforward^[34]^. Parentage assignments were successful for > 97% of the seeds in each crop; the few unassigned seeds are likely contaminations that occurred during cone processing. We detected contributions from rootstock and unknown parents in five of the six sampled orchards, reflecting difficulties in tracking minor changes in orchard plantations made by management. The detected BPC ranged from 11.9% to 86.5% in pine, and 3.6% to 26.9% in spruce. Wide variations in BPC rates have been reported previously for Scots pine orchards (see Torimaru et al.^[15]^ for summary). For Norway spruce, 70% BPC was reported for an orchard in Finland^[19]^, 18%−29% in crops from two orchards in Norway^[20]^, and 55%-61% in an orchard in Poland^[50]^. These studies utilized either allozyme or SSR markers. The limited number of marker loci used in these studies, together with the difficulty of correctly scoring null alleles in these marker systems may have constrained assignment precision (see Funda et al.^[16]^ for possible issues).

Apart from the marker systems, there have been a plethora of studies that discuss possible factors affecting BPC, including distance of isolation from conspecific forests, flowering asynchrony, orchard age and pollen production of orchard parents, and environmental conditions of orchard location^[2]^. In this study, we observed a clear difference in BPC rates between pine and spruce orchards. This is most likely due to differences in their pollen dispersal range and pollen fecundity. Compared to Scots pine, Norway spruce flowers less often and not as abundantly and pollen grains are heavier and migrate shorter distances^[51]^. In this regard, pollen contamination is a greater challenge for Scots pine seed orchard management.

We also observed differences between pine and spruce in BPC rates with increasing orchard age. Pollen contamination is expected to be relatively high in young orchards because of low internal pollen production. However, examining the effects of orchard age without adjusting for climate at orchard location could be misleading. Orchards in the harsher climates further north reach maturity later, resulting in high BPC for a longer period before orchards reach peak pollen production. The crops from the youngest orchards of both species show the highest levels of BPC, although the level of BPC in the Bredinge 2000 spruce crop is likely an over-estimation. In addition, we expect that the Bredinge 2000 crop (age 17) was produced when pollen production in Bredinge was substantially greater than in Klocke at age 15 (assuming seed production also reflects pollen production, Table 1), given the more southerly location and milder climate in Bredinge. We observed a substantial reduction of BPC in mature pine orchards, but not among spruce orchards. Ignoring the Bredinge 2000 crop, the remaining three spruce crops were between 20−43 years of age, but their BPC were relatively stable, 8.7%−3.6%, in stark contrast to the much wider range of 86.5%−11.9% in pine crops. This suggests a much more fluctuating pollen contribution from orchard trees and conspecific stands in pine orchards over time. Factors that might explain these observations include temporal differences in phenology, which could differ over years depending on among-year variation in degree-day sum and wind speeds during the pollination period^[10]^. Although we also expect these factors to influence spruce BPC, their impact may be lower than in pine orchards because of the infrequent flowering and low BPC in spruce.

Flowering synchrony among parents is a key condition to ensure random mating and equal genetic contribution to progeny. In both Norway spruce and Scots pine orchards, flowering is generally synchronized^[17,52]^. The observed high variance in parental contribution to each crop thus suggests substantial variation in fecundity among parents. In addition, uneven representation of parents, such as the linear deployment design used in the Västerhus orchard, with more ramets for parents of high breeding value, further increases the variance in parental contribution^[8]^. The dominating parents are similar over years in pine (Klocke in Fig. 4 and Västerhus^[16]^, in contrast to the larger between-year variation in spruce (see Maglehem in Fig. 4). These results support previous findings which suggest that variation among parents in response to environmental cues for flowering has a genetic component within Scots pine and Norway spruce orchards^[8,10,17,20]^. The *ex situ* production of seeds may thus suffer from unpredictable year to year variation in genetic composition in orchards due to variable BPC and parental contributions.

Silviculture management practices such as thinning, which occurred before the pollination year of the third crop in Klocke (Klocke 2008), may have lowered the overall orchard pollen production and allowed more external pollen flow into the pine orchard when open corridors were created. This is the likely reason for the rebound of BPC from 12% in Klocke 1996 to 48% in Klocke 2008. In contrast, BPC in the spruce orchard Maglehem increased only slightly after thinning^[53]^, again indicating different pollination dynamics between the two species. Because of these thinning events, the natural progression of diversity and BPC with age was interrupted, and we therefore did not observe significant correlations between orchard age and diversity parameters (Table 3). Given more data that consider trade-offs between seed production, BPC, diversity and breeding gain, optimum ages for thinning or other orchard operations could be established for pine and spruce^[54,55]^.

Pollen flow from unimproved natural stands to seed orchards reduces breeding gain of orchard crops proportional to the BPC level in each crop. In the Swedish tree breeding and regeneration material selection system Plantval (www.skogforsk.se/produkter-och-evenemang/verktyg/plantval/), 40% BPC is assumed for mature Scots pine orchards and 0% in spruce orchard. From our results, it seems spruce orchards have an overall low BPC 4%−9% if we disregard Bredinge 2000. Thus reduction of BPC on the expected breeding gain (assuming 0% BPC) in each crops is minor, only 1% (Table 4). We suggest a 10% BPC in Plantval for spruce would be a good approximation. The gain reduction for Scots pine crops is more variable due to the large range in BPC (12%−87%, Table 4). Relative to the assumed 40% BPC in Plantval, very early crops would far exceed this level, and thus have a lower gain than expected, while under full pollen production, the observed BPC can be much lower than 40%, thus a under estimation of gain. This is exemplified by Klocke crops; the very early crop from 1985 is estimated to have a gain 4% lower than expected, while in the 1996 crop the estimated gain is 3% higher than expected (Table 4). Our results suggest that pine orchards at full production age are either close to 40% BPC or lower, thus the 40% default setting in Plantval is a reasonable approximation. However, for advanced orchards with expected breeding gain as high as 25%, elevated BPC can lead to substantial reduction in gain. Thus systematic assessment of BPC in pine orchards in particular, should become a routine procedure in seed orchard management to guide dynamic deployment of forest regeneration materials.

**Table 4:** Impact of background pollen contamination (BPC) on expected breeding gain.

Orchard	Plantval			Under the observed BPC		
	Exp. gain	BPC		Crop	BPC	Gain
**Norway spruce**						
Lillpite	10%	0%		Lillpite1983	8.7%	9.1%
Bredinge	15%	0%		Bredinge 2000	26.9%	11.0%
Maglehem	10%	0%		Maglehem 1993	3.6%	9.6%
				Maglehem 2000	6.6%	9.3%
**Scots pine**						
Klocke	5%	40%		Klocke 1985	86.5%	1.1%
Västerhus	19%	40%		Klocke 1996	11.9%	7.3%
Lilla istad	11%	40%		Klocke 2008	48.2%	4.3%
				Västerhus 2014	31.1%	21.8%
				Lilla Istad 2007	43.3%	10.4%

## CONCLUSIONS

This study represents the first large scale comparative analysis of mating system and genetic diversity in Scots pine and Norway spruce seed orchards. We detected a slight reduction in genetic diversity in the seed crops compared to natural stands in both species, but no significant signals of inbreeding in any orchard crops. We see a significant positive correlation between BPC and observed heterozygosity *H*_O_, but correlation is not evident between census number of orchard parents and *H*_O_, suggesting an orchard and site specific diversity. The two species showed clear differences in the levels of *H*_O_ and BPC, with lower *H*_O_ and BPC, and less variation in BPC over orchard age in spruce. Thus, the genetic quality of Norway spruce orchard crops appeared to be less affected by BPC compared to Scots pine orchards. However, high fecundity variation among parents and between years in Norway spruce could lead to unexpected genetic compositions. Evaluation of BPC should become a routine procedure in orchard management to monitor the progression of breeding gain and adaptation of orchard crops. Our results will serve as a valuable reference for setting up optimal orchard management strategies for each species.

## SUPPLEMENTARY DATA

Supplementary data to this article can be found online.
