# Supplementary material for: Genetic diversity and background pollen contamination in Norway spruce and Scots pine seed orchard crops
Source: For Res (Fayettev). 2022 Jun 1;2:8. doi: 10.48130/FR-2022-0008 (PMC11524256; doi:10.48130/FR-2022-0008)

Genetic diversity and background pollen contamination in Norway spruce and Scots pine seed orchard crops

Alisa Heuchel, David Hall, Wei Zhao, Jie Gao, Ulfstand Wennström, Xiao-Ru Wang

Supplementary material

Deployment areas of the seed orchards. The upper panels are three Scots pine seed orchards, and lower panels are three Norway spruce seed orchards. The location of each orchard is marked as red dot.

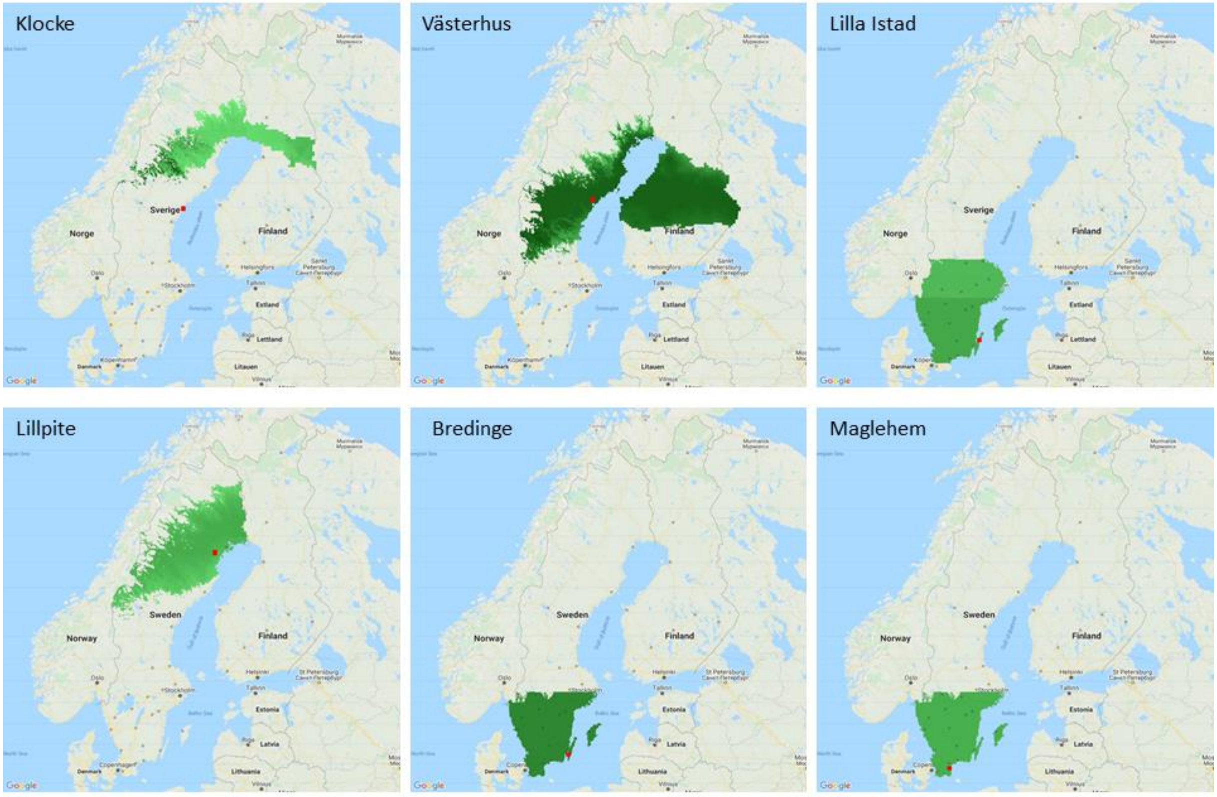

Supplement: Supplementary file 1 — Supplementary data to this article can be found online. [file FR-2022-0008-Suppl-FigureS1.pdf]
